# Improved in-vivo airway gene transfer via magnetic-guidance, with protocol development informed by synchrotron imaging

**DOI:** 10.1038/s41598-022-12895-x

**Published:** 2022-05-30

**Authors:** Martin Donnelley, Patricia Cmielewski, Kaye Morgan, Juliette Delhove, Nicole Reyne, Alexandra McCarron, Nathan Rout-Pitt, Victoria Drysdale, Chantelle Carpentieri, Kathryn Spiers, Akihisa Takeuchi, Kentaro Uesugi, Naoto Yagi, David Parsons

**Affiliations:** 1 Robinson Research Institute, University of Adelaide, Adelaide, SA 5001 Australia; 2 Adelaide Medical School, University of Adelaide, Adelaide, SA 5001 Australia; 3 Respiratory and Sleep Medicine, Women’s and Children’s Hospital, 72 King William Road, North Adelaide, SA 5006 Australia; 4 School of Physics and Astronomy, Monash University, Clayton, VIC 3800 Australia; 5 Deutsches Elektronen-Synchrotron DESY, Notkestr. 85, 22607 Hamburg, Germany; 6 SPring-8 Synchrotron, Japan Synchrotron Radiation Institute, Kouto, Hyogo Japan

**Keywords:** Genetic transduction, X-rays

## Abstract

Gene vectors to treat cystic fibrosis lung disease should be targeted to the conducting airways, as peripheral lung transduction does not offer therapeutic benefit. Viral transduction efficiency is directly related to the vector residence time. However, delivered fluids such as gene vectors naturally spread to the alveoli during inspiration, and therapeutic particles of any form are rapidly cleared via mucociliary transit. Extending gene vector residence time within the conducting airways is important, but hard to achieve. Gene vector conjugated magnetic particles that can be guided to the conducting airway surfaces could improve regional targeting. Due to the challenges of in-vivo visualisation, the behaviour of such small magnetic particles on the airway surface in the presence of an applied magnetic field is poorly understood. The aim of this study was to use synchrotron imaging to visualise the in-vivo motion of a range of magnetic particles in the trachea of anaesthetised rats to examine the dynamics and patterns of individual and bulk particle behaviour in-vivo. We also then assessed whether lentiviral-magnetic particle delivery in the presence of a magnetic field increases transduction efficiency in the rat trachea. Synchrotron X-ray imaging revealed the behaviour of magnetic particles in stationary and moving magnetic fields, both in-vitro and in-vivo. Particles could not easily be dragged along the live airway surface with the magnet, but during delivery deposition was focussed within the field of view where the magnetic field was the strongest. Transduction efficiency was also improved six-fold when the lentiviral-magnetic particles were delivered in the presence of a magnetic field. Together these results show that lentiviral-magnetic particles and magnetic fields may be a valuable approach for improving gene vector targeting and increasing transduction levels in the conducting airways in-vivo.

## Introduction

Cystic fibrosis (CF) is caused by variants in a single gene called the CF transmembrane conductance regulator (*CFTR*). The CFTR protein is an ion channel that is present in many epithelial cells throughout the body, including the conducting airways, the primary site of CF pathogenesis. The CFTR defect results in abnormal water transport, dehydrating the airway surface and reducing the depth of the airway surface liquid (ASL) layer. This also compromises the ability of the mucociliary transit (MCT) system to clear inhaled particulates and pathogens from the airways. Our goals are to develop a lentiviral (LV) gene therapy to deliver a correct copy of the CFTR gene and improve ASL, MCT and lung health, and to continue to develop novel techniques able to measure these parameters in-vivo^1^.

LV vectors are one of the lead candidates for a CF airway gene therapy, primarily because they can permanently integrate the therapeutic gene into airway basal cells, a stem cell of the airway^2^. This is important because they could then produce life-long benefit by differentiating into functional gene corrected CF-relevant airway surface cells that restore normal hydration and mucus clearance^1^. LV vectors should target the conducting airways, since this is the location where CF lung disease initiates. Delivering vectors deeper into the lung might produce alveolar transduction, but this does not offer any therapeutic benefit for CF. However, fluids such as gene vectors naturally move to the alveoli during inspiration after delivery^3,4^, and therapeutic particles are rapidly cleared to the mouth via MCT. LV transduction efficiency is directly related to the length of time the vector is retained next to target cells to allow cellular uptake—the “residence time”^5^—which is readily reduced by typical regional airflows, and the coordinated particle mucus capture and MCT. For CF, the ability to extend the LV residence time within the conducting airways is important to achieve high levels of transduction in that region, but so far has been challenging.

To overcome this barrier we propose that LV magnetic particles (MP) may be helpful in two complementary ways. First, they could be magnetically guided to the conducting airway surfaces to improve targeting and help the gene vector particles reside in the desired conducting airway regions; and secondly, the magnetic force may also assist the LV vector particles to move through the surface layers (mucus, secretions and ASL) to the cell layer^6^. MP have been extensively used in cancer therapy as a targeted drug-delivery vehicle when conjugated with antibodies, chemotherapeutics, or other small molecules that attach to cell membranes or bind to relevant cell surface receptors and accumulate at the tumor site in the presence of a static magnetic field^7^. Other “hyperthermic” techniques are designed to heat up the MP when exposed to an oscillating magnetic field, thereby destroying the tumor cells. The principle of magnetofection, in which the magnetic field is used like a transfection agent to enhance DNA transfer to cells, is commonly employed in-vitro for difficult-to-transduce cell lines using a range of non-viral and viral gene vectors^8^. The effectiveness of LV magnetofection has been established, with LV-MP delivered to a human bronchial epithelial cell line in-vitro in the presence of a static magnetic field increasing transduction efficiency by 186-fold compared to LV vector alone^9^. LV-MP have also been applied to in-vitro CF models, with magnetofection increasing LV transduction of air liquid interface cultures in the presence of CF sputum by 20-fold^10^. However, in-vivo magnetofection of organs has received comparatively little attention, and has only been evaluated in a small number of animal studies^11–15^, with even fewer specifically in the lung^16,17^. Nonetheless, the opportunity for magnetofection in CF lung treatment is clear. Tan et al. (2020) noted that “Proof-of-concept studies of effective magnetic nanoparticle lung delivery will pave the way for future *CFTR* inhalation strategies to improve clinical outcomes in patients with CF”^6^.

The behaviour of small magnetic particles on the airway surface in the presence of an externally applied magnetic field is difficult to visualise and study, and therefore poorly understood. In other studies we have developed synchrotron propagation-based phase contrast X-ray imaging (PB-PCXI) methods for non-invasively visualising and quantifying tiny in-vivo changes in the ASL depth^18^ and of MCT behaviour^19,20^, to directly measure airway surface hydration and for use as early indicators of treatment effectiveness. Further, our MCT assessment method uses 10–35 µm diameter particles composed of alumina or high refractive index glass as MCT markers that are visible using PB-PCXI^21^. Both techniques are adaptable to visualisation of a range of particle types, including MP.

Due to their high spatial and temporal resolution, our PB-PCXI-based ASL and MCT analysis techniques are ideal for application to the examination of the dynamics and patterns of individual and bulk particle behaviour in-vivo, to aid our understanding and optimisation of MP technologies for gene delivery. The approach we employ here follows on from studies we performed using the SPring-8 BL20B2 beamline, where we visualised fluid motion following sham vector dose delivery into mouse nasal and lung airways to help explain the patterns of non-uniform gene expression that we observe during our gene vector dosing animal studies^3,4^.

The aim of the present study was to use synchrotron PB-PCXI to visualise the in-vivo motion of a range of MP in the trachea of live rats. These PB-PCXI imaging studies were designed to test a range of MP, magnetic field strengths, and locations to establish their effects on MP motion. We hypothesised that the externally applied magnetic field would aid the delivered MP to remain in or move into the targeted region. These studies also enabled us to identify the magnet configuration that maximised the number of particles retained within the trachea following deposition. In a second series of studies we sought to use that optimal configuration to demonstrate the transduction patterns that result from LV-MP delivery into rat airways in-vivo, based on the hypothesis that delivery of LV-MP in the presence of an airway-targeted magnetic field would result in improved LV transduction efficiency.

## Methods

All animal studies were performed in accordance with protocols approved by the University of Adelaide (M-2019-060 and M-2020-022) and SPring-8 Synchrotron animal ethics committees. The experiments were conducted in accordance with the ARRIVE guidelines.

### X-ray imaging studies

#### Imaging configuration

All X-ray imaging was conducted on the BL20XU beamline at the SPring-8 Synchrotron in Japan using a similar setup to previously described^21,22^. Briefly, the experimental hutch was located 245 m from the Synchrotron storage ring. A sample to detector distance of 0.6 m was used for the particle imaging studies, and 0.3 m for the in-vivo imaging studies, to generate phase contrast effects. A monochromatic beam energy of 25 keV was used. Images were captured using a high-resolution X-ray converter (SPring-8 BM3) coupled to a sCMOS detector. The converter used a 10 µm thick scintillator (Gd_3_Al_2_Ga_3_O_12_) to convert X-rays to visible light, which was then directed to the sCMOS sensor using a × 10 microscope objective lens (NA 0.3). The sCMOS detector was an Orca-Flash4.0 (Hamamatsu Photonics, Japan) with an array size of 2048 × 2048 pixels and a 6.5 × 6.5 µm native pixel size. This setup resulted in an effective isotropic pixel size of 0.51 µm and a field of view of approximately 1.1 mm × 1.1 mm. An exposure length of 100 ms was chosen to maximise the signal to noise ratio of the magnetic particles outside and within the airway, while also minimising motion artifacts from respiration. For the in-vivo studies a fast X-ray shutter was placed into the X-ray path to limit the radiation dose by blocking the X-ray beam between exposures.

#### Magnetic particle imaging

LV vectors were not used in any SPring-8 PB-PCXI imaging studies because the BL20XU imaging hutch is not Biosafety Level 2 certified. Instead, we chose a range of MP with well characterised properties available from two commercial suppliers—to cover a range of sizes, materials, iron concentrations, and applications—to first understand how the magnetic field affects MP motion within glass capillary tubes, and then on live airway surfaces. The MP ranged in size from 0.25 to 18 μm and were made from a range of materials (See Table 1), however the composition of each sample, including the size of the magnetic particles within the MP, was not known. Based on our extensive MCT studies^19–21,23,24^ we expected that MP as small as 5 μm would be visible on the tracheal airway surface, with visibility enhanced by MP motion, for example, seen by subtracting sequential frames. Individual 0.25 μm sized MP were smaller than the resolution of the imaging setup, but it was predicted that their bulk contrast and the motion of the surface fluid in which they resided after deposition would be detectable by PB-PCXI.

**Table 1:** Properties of the six particles tested at SPring-8 BL20XU.

Sample ID	Supplier	Cat No	Description	Composition
MP1	Corpuscular (NY, USA)	106531-10	COOH Magnetic polystyrene Diameter = 18 μm (10 ml, 1%)	10–12% Fe_3_O_4_
MP2		106,409–10	COOH Magnetic polystyrene Diameter = 0.25 μm (10 ml, 1%)	
MP3		144416-05	C-MAGSIO-0.25COOH Diameter = 0.25 μm (5 ml, 2.5%)	10–15% Fe_3_O_4_
MP4		144516-05	C-MAGSIO-1.0COOH Diameter = 0.9 μm (5 ml, 5%)	
MP5		New MagSIO	C-MAGSIO-0.25COOH Diameter = 0.25 μm (5 ml, 5%)	98% Fe_3_O_4_
MP6	OZ Biosciences (France)	CombiMag	Iron oxide, coated with proprietary cationic molecules. Association with the LV vector is achieved by salt-induced colloidal aggregation and electrostatic interaction Diameter = 0.16–0.2 μm	Unknown (5 × 10^9^ to 5 × 10^10^ particles/ml)

**Figure 1:**
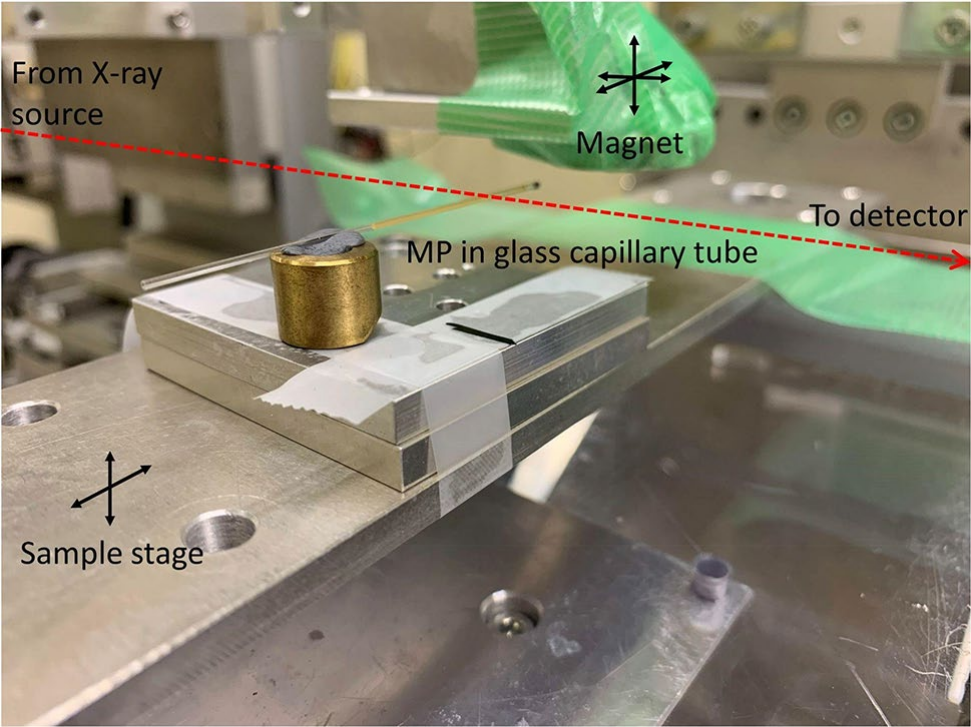
in-vitro imaging setup with a MP sample contained within a glass capillary tube on the sample x–y translation stage. The path of the X-ray beam is marked with a red dashed line.

Samples of each MP from Table 1 were prepared in 20 μl glass capillary tubes with an internal diameter of 0.63 mm (Drummond Microcaps, PA, USA). The Corpuscular particles were supplied in water, and the CombiMag particles in the manufacturer’s proprietary fluid. Each tube was half filled with fluid (approximately 11 μl) and placed onto a sample holder (see Fig. 1). The glass capillaries were individually placed horizontally on the sample stage in the imaging hutch and the edge of the fluid was located. A 19 mm diameter (28 mm length) nickel-cased rare-earth neodymium iron boron (NdFeB) magnet (N35, cat # LM1652, Jaycar Electronics, Australia) with a remanent magnetisation of 1.17 Tesla was attached to a separate translation stage to enable its location to be remotely altered during imaging. X-ray image acquisition started while the magnet was located approximately 30 mm above the sample, with images acquired at a rate of 4 frames per second. During imaging the magnet was brought closer to the glass capillary tube (~ 1 mm away), and then translated along the tube to allow the effects of field strength and position to be assessed.

#### in-vivo imaging studies

**Figure 2:**
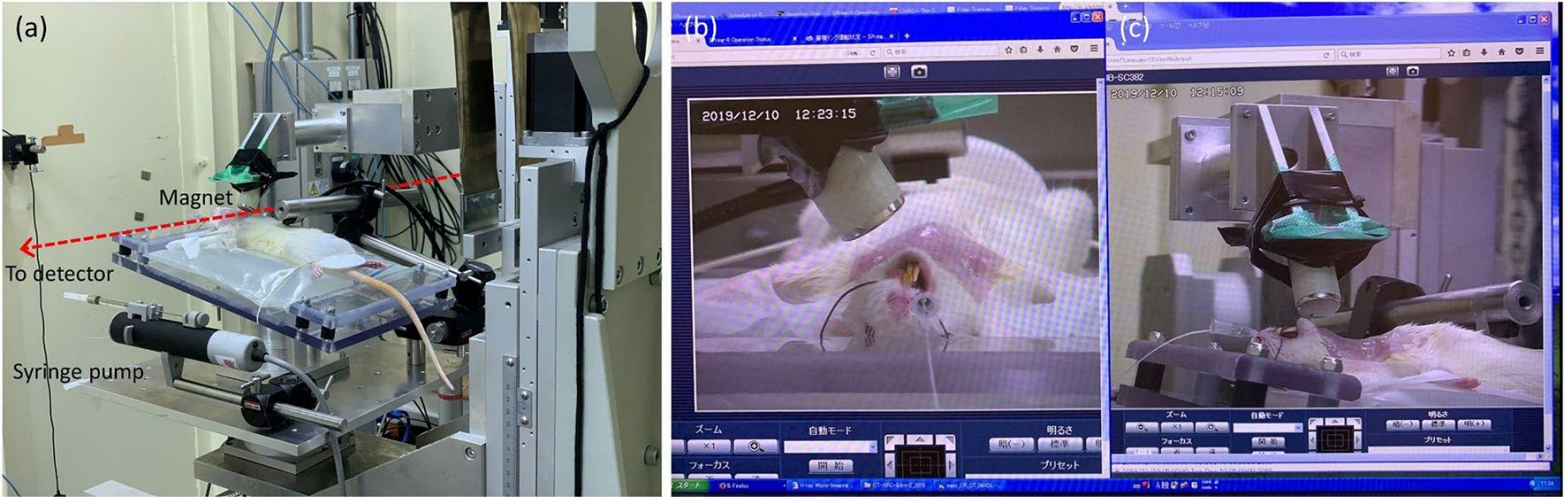
(**a**) in-vivo imaging setup in the SPring-8 imaging hutch, with the path of the X-ray beam marked with a red dashed line. (**b**,**c**) The magnet positioning over the trachea was performed remotely using two orthogonally mounted IP cameras. On the left-hand side of the screen image the wire loop securing the head, and the delivery cannula in position within the ET tube, can be seen.

Once the in-vitro visibility of the MP was determined, a selection of these were tested in-vivo in wild-type female albino Wistar rats (~ 12 weeks of age, ~ 200 g). Rats were anaesthetised with a mixture of 0.24 mg/kg of medetomidine (Domitor®, Zenoaq, Japan), 3.2 mg/kg midazolam (Dormicum®, Astellas Pharma, Japan), and 4 mg/kg butorphanol (Vetorphale®, Meiji Seika Pharma, Japan) via intraperitoneal injection. Once anaesthetised, the rats were prepared for imaging by removal of fur from around the trachea, insertion of an endotracheal tube (ET; 16 Ga i.v. cannula, Terumo BCT), and securing them supine to a custom-designed imaging board containing a heat pack to maintain body temperature^22^. This imaging board was then attached to the sample translation stage in the imaging hutch at a slight incline, to align the trachea horizontally in the X-ray images, as shown in Fig. 2a.

A remotely actuated syringe pump system (UMP2, World Precision Instruments, Sarasota, FL) utilising a 100 μl glass syringe was connected via a 30 Ga needle to PE10 tubing (OD 0.61 mm, ID 0.28 mm). The tubing was marked to ensure that the tip was at the correct location within the trachea when it was inserted into the ET tube. Using the micropump, the syringe piston was withdrawn while the tube tip was immersed in the MP sample to be delivered. The loaded delivery tubing was then inserted into the endotracheal tube, with the tip placed to be within what we expected to be the strongest part of the applied magnetic field. Image acquisition was controlled using a respiration detector connected to our Arduino-based timing box, with all signals (e.g. temperature, respiration, shutter open/close, and image acquisition) recorded using a Powerlab and LabChart (AD Instruments, Sydney, Australia)^22^. Two IP cameras (Panasonic BB-SC382) located at approximately 90° to each other were used to monitor the location of the magnet relative to the trachea during imaging, when the imaging enclosure was not accessible (Fig. 2b,c). To minimise motion artefacts, one image was acquired per breath, during the end-of-expiration flow plateau.

**Figure 3:**
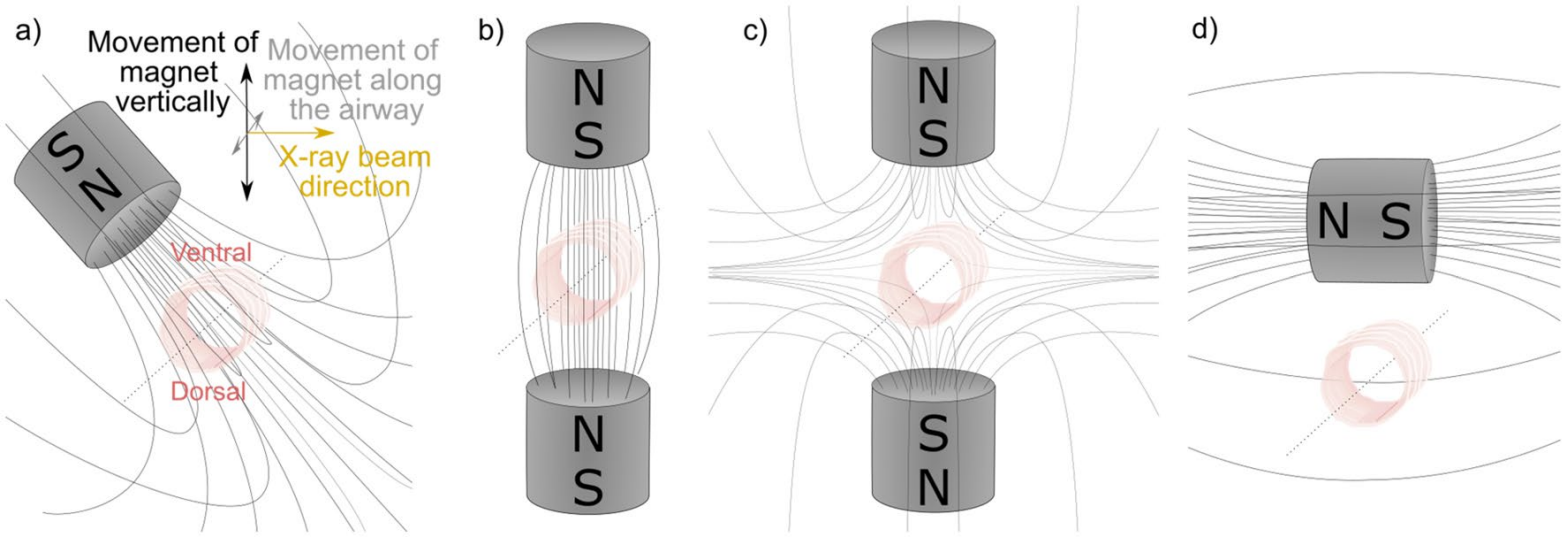
Magnet configurations for in-vivo imaging (**a**) Single magnet above trachea at ~ 30° angle, (**b**) two magnets set up to attract, (**c**) two magnets set up to repel, (**d**) a single magnet above and perpendicular to the trachea. The viewer looks down through the trachea from the mouth towards the lungs, with the X-ray beam passing into the rat’s left side and out its right side. The magnet was moved either along the length of the airway, or left and right above the trachea in the direction of the X-ray beam.

The magnet was attached to a second stage that could be remotely positioned from outside the imaging enclosure. A variety of magnet locations and configurations were tested, including: mounted at a ~ 30° angle above the trachea (the configuration shown in Figs. 2a and 3a); one magnet above the animal and the other below with poles set up to attract (Fig. 3b); one magnet above the animal and the other below, with poles set up to repel (Fig. 3c); and one magnet above and perpendicular to the trachea (Fig. 3d). Once the animal and magnet were configured and the MP to be tested were loaded in the syringe pump, a 50 μl dose was delivered at a rate of 4 μl/sec while images were acquired. The magnet was then moved back and forth, either along the trachea, or transversely across the trachea while continuing to acquire images.

We also sought to determine the visibility and behaviour of the particles in the airways when confounding respiratory and cardiac motion were absent. Therefore, at the end of the imaging period animals were humanely killed by pentobarbital overdose (Somnopentil, Pitman-Moore, Washington Crossing, USA; ~ 65 mg/kg i.p.). Some animals were left in place on the imaging platform, and once respiration and heartbeat ceased the imaging procedure was repeated, with an additional dose of MP added if none remained visible on the airway surface.

Acquired images were flat- and dark-field corrected and then assembled into movies (20 frames per second; 15–25 × normal speed depending on respiratory rate) using custom scripts written in MATLAB (R2020a, The Mathworks).

### in-vivo gene vector studies

All LV gene vector delivery studies were performed at the University of Adelaide laboratory animal research facility and were designed to utilise the findings from the SPring-8 experiments, to assess whether delivery of LV-MP in the presence of a magnetic field enhances in-vivo gene transfer. To assess the effect of the MP and magnetic field, two groups of animals were treated: one dosed with LV-MP with the magnet in place, and the other a control group that received LV-MP without the magnet.

#### Gene vector production

The LV gene vector was produced using previously described methods^25,26^. The LacZ vector expressed a nuclear-localised β-galactosidase gene driven by the constitutive MPSV promoter (LV-LacZ), which produces a blue reaction product in transduced cells that is visible in lung tissue *en face* and in histological sections. Titering was performed in cell culture by manually counting the number of LacZ-positive cells with a haemocytometer to calculate the titre in TU/ml. The vector was stored frozen at − 80 °C, thawed just prior to use, and conjugated to the CombiMag by mixing in a 1:1 ratio and incubating on ice for at least 30 min prior to delivery.

#### Gene vector delivery

Normal Sprague Dawley rats (n = 3/group, ~ 2–3 months age) were anaesthetised with a mixture of 0.4 mg/kg medetomidine (Domitor, Ilium, Australia) and 60 mg/kg ketamine (Ilium, Australia) by intraperitoneal (i.p.) injection, and were non-surgically orally intubated with a 16 Ga i.v. cannula. To ensure that the tracheal airway tissue was receptive to LV transduction^27^, it was conditioned using our previously-described mechanical perturbation protocol, in which the tracheal airway surface was rubbed axially with a wire basket (N-Circle, Nitinol Tipless Stone Extractor NTSE-022115-UDH, Cook Medical, USA) for 30 s^28^. LV-MP tracheal dosing was then performed in a biosafety cabinet ~ 10 min after perturbation.

**Figure 4:**
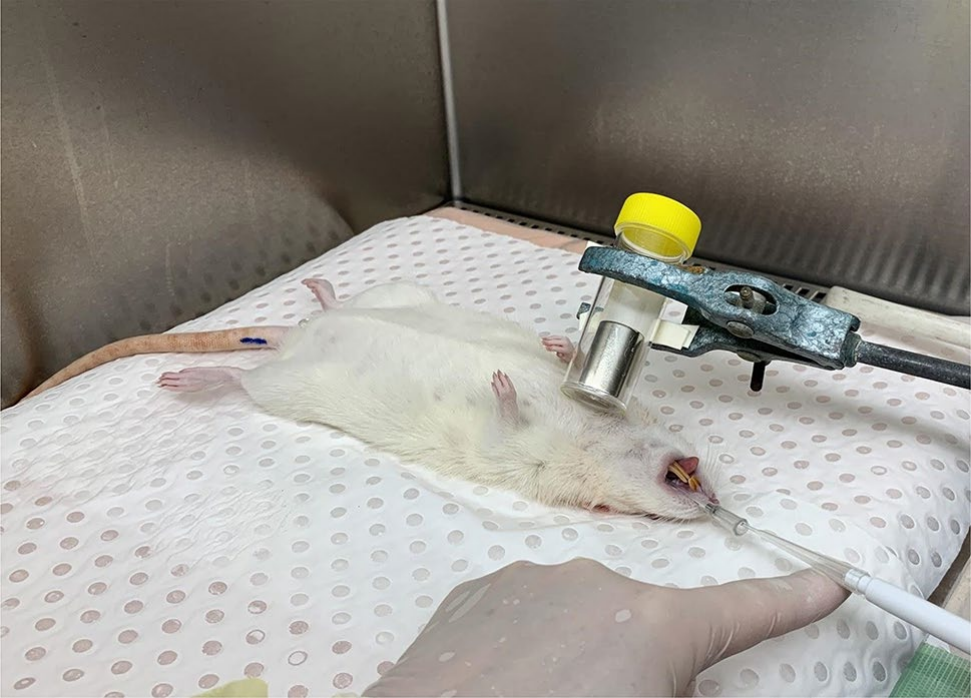
LV-MP delivery setup in a biosafety cabinet. The light grey Luer hub of the ET tube can be seen projecting from the mouth, with the pipette gel-tip shown inserted into the trachea to the desired depth via the ET tube.

The magnetic field used in this experiment was configured in a similar manner as for the in-vivo X-ray imaging studies, with the same magnet held above the trachea using a retort stand clamp (Fig. 4). A 50 μl volume (2 × 25 μl aliquots) of LV-MP was delivered to the trachea (n = 3 animals) using a pipette containing a gel tip as previously described^25^. The control group (n = 3 animals) received the same LV-MP, but the magnet was not used. After fluid delivery was completed, the cannula was removed from the ET tube, and the animal was extubated. The magnet remained in place for 10 min, after which it was removed. Rats received a subcutaneous dose of meloxicam (1 ml/kg) (Ilium, Australia) and then anaesthesia was reversed with an i.p. injection of 1 mg/kg atipamezole hydrochloride (Antisedan, Zoetis, Australia). Rats were kept warm and monitored until recovery from anaesthetic was complete.

#### Gene expression assessment

One week after the LV-MP dosing procedure, the animals were humanely killed via 100% CO_2_ inhalation, and LacZ expression was assessed using our standard X-gal processing method^29^. The three most-caudal cartilage rings were removed to ensure that any mechanical damage or fluid retention from the endotracheal tube placement was not included in the analysis. Each trachea was cut lengthwise to produce two halves for analysis, and these were pinned out to display the lumenal surfaces using Minutien pins (Fine Science Tools) into a dish containing silicone elastomer (Sylgard, Dow Inc). The distribution and pattern of transduced cells was confirmed by *en face* photography using a Nikon microscope (SMZ1500) with a DigiLite Camera and TCapture software (Tucsen Photonics, China). Images were acquired at × 20 magnification (the highest setting that encompassed the full width of the trachea) and the entire length of the trachea was imaged stepwise, ensuring that there was sufficient overlap between each image to enable image “stitching”. Images from each trachea were then assembled into a single composite image using Image Composite Editor v2.0.3 (Microsoft Research), utilising the planar motion algorithm. The area of LacZ expression within the tracheal composite images from each animal were quantified using an automated MATLAB script (R2020a, MathWorks) as previously described^28^, using settings of 0.35 < Hue < 0.58, Saturation > 0.15, and Value < 0.7. A mask was manually generated for each composite image in GIMP v2.10.24 by tracing the outline of the tissue, to enable the tissue area to be determined and prevent any false detections from outside the tracheal tissue. The stained area from all the composite images from each animal was summed to produce a total staining area for that animal. The stained area was then divided by the total mask area to produce a normalised area.

Each trachea was paraffin embedded and 5 μm sections were cut. Sections were counterstained with neutral fast red for 5 min and images were acquired with a Nikon Eclipse E400 microscope, DS-Fi3 camera and NIS elements capture software (version 5.20.00).

### Statistical analyses

All statistical analyses were performed in GraphPad Prism v9 (GraphPad Software, Inc.). Statistical significance was set at *p* ≤ 0.05. Normality was verified using the Shapiro–Wilk test, and the LacZ staining differences assessed using an unpaired t-test.

## Results

### X-ray imaging studies

#### Magnetic particle imaging

**Figure 5:**
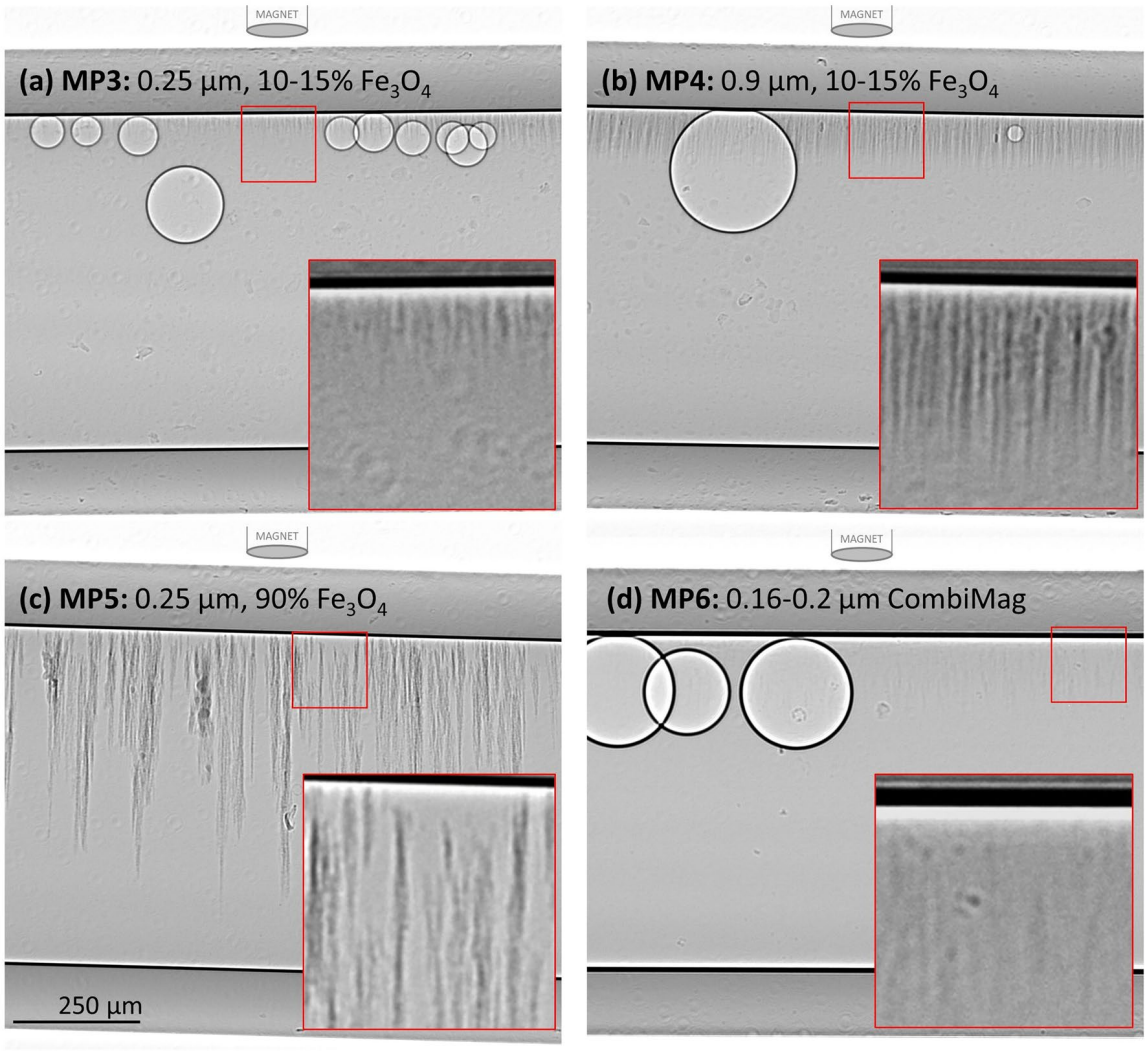
The visibility of the MP under PCXI varied markedly between the samples. (**a**) MP3, (**b**) MP4, (**c**) MP5 and (**d**) MP6. All images displayed here were taken with the magnet located ~ 10 mm directly above the capillary tube. The distinct large circular shapes are air bubbles trapped in the capillary tube, and clearly show the black-white edges characteristic of phase contrast imaging. Red boxes contain a contrast-enhanced enlargement. Note that the diameter of the magnet schematic in all Figures is not to scale and is ~ 100 × larger than shown.

The six MP described in Table 1 were all examined with PCXI, with the visibility described in Table 2. The two polystyrene MP (MP1 and MP2; 18 μm and 0.25 μm, respectively) were not visible under PCXI, but the remaining samples could all be identified (examples shown in Fig. 5). MP3 and MP4 (10–15% Fe3O4; 0.25 μm and 0.9 μm, respectively) were weakly visible. Despite containing some of the smallest particles tested, MP5 (98% Fe3O4; 0.25 μm) was the most visible. MP6, the CombiMag product, was very hard to detect. In all cases our ability to detect the MP was dramatically enhanced by translating the magnet back and forth parallel to the capillary. When the magnet was located away from the capillary tube the particles extended in long strings, but when the magnet was brought closer and the magnetic field strength increased the strings shortened as the particles migrated toward the top surface of the capillary (See Supplementary Video S1: MP4), increasing the density of particles at that surface. In contrast, when the magnet was moved away from the capillary, reducing the field strength, the MP reordered into long strings that extended away from the upper capillary surface (See Supplementary Video S2: MP4). The particles continued to move for a short period after the magnet stopped moving as they reached an equilibrium position. As the MP moved toward and away from the top surface of the capillary the magnetic particles often dragged debris along with them through the fluid.

**Table 2:** Visibility of the tested magnetic particles using PCXI at SPring-8 BL20XU.

Sample ID	Brief description	Visible in-vitro	Visible in-vivo	in-vivo magnet configurations tested
MP1	18 μm Polystyrene	No	Not tested	–
MP2	0.25 μm Polystyrene	No	Not tested	–
MP3	0.25 μm 10–15% Fe3O4	Weakly	No	–
MP4	0.9 μm 10–15% Fe3O4	Weakly	No	–
MP5	0.25 μm 98% Fe3O4	Strongly	Weakly	a, b, c, d
MP6	CombiMag	Weakly	No	–

**Figure 6:**
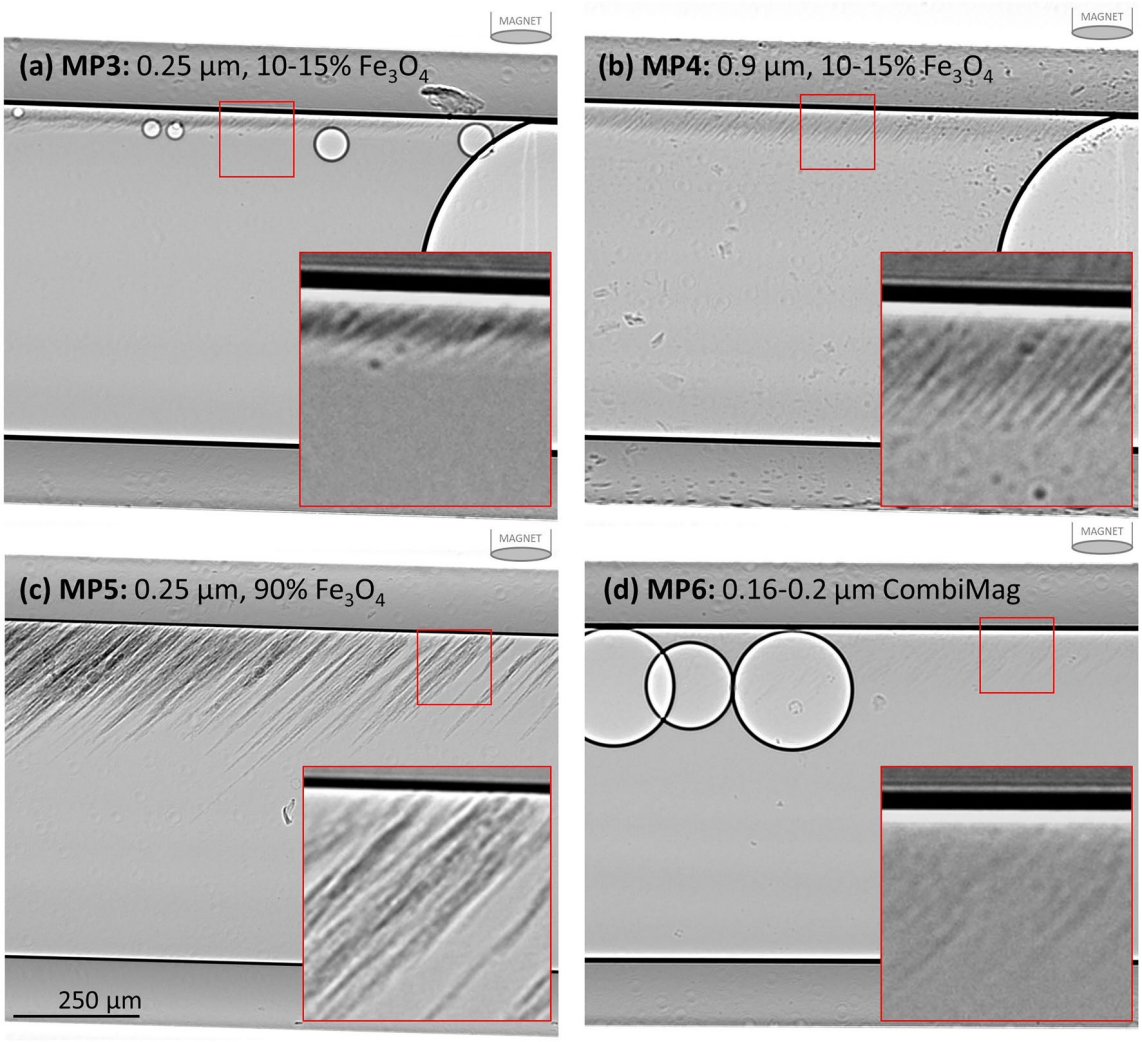
As the magnet was translated to the right above the capillary tube, the angle of the MP strings changed. (**a**) MP3, (**b**) MP4, (**c**) MP5 and (**d**) MP6. Red boxes contain a contrast-enhanced enlargement. Note that the supplementary videos are informative as they reveal important particle structure and dynamic information that cannot be visualised in these static images.

When the magnet was translated along the top of the capillary tube to the left and right, the angle of the MP strings changed to align towards the magnet (See Fig. 6), tracing out the magnetic field lines. For MP3-5, the strings reached a threshold angle after which the particles were dragged along the top surface of the capillary tube. This often caused the MP to aggregate into larger groups that settled close to where the magnetic field was strongest (See supplementary Video S3: MP5). This was also particularly noticeable when imaging close to the end of the capillary, which resulted in the MP bunching up and becoming concentrated at the fluid to air interface. The particles in MP6 were more difficult to discern than MP3-5, and when the magnet was moved along the capillary these particles were not dragged along, but instead the MP strings dissociated, with the particles remaining in the field of view (See Supplementary video S4: MP6). In some cases, when the applied magnetic field was reduced by moving the magnet a very long distance away from the imaging location any remaining MP slowly dropped to the bottom surface of the tube under gravity, while remaining in strings (See Supplementary Video S5: MP3).

#### in-vivo imaging studies

Our testing showed that slowly translating the magnet back and forth along the trachea aided in the visualisation of the MP against the complex moving background in-vivo. Since the polystyrene beads (MP1 and MP2) were not visible in the capillary tube they were not tested in-vivo. Each of the remaining four MP were tested in-vivo, with the magnet long-axis configured above the trachea at an angle of ~ 30° to vertical (See Figs. 2b and 3a) as this resulted in longer MP strands and stronger effects than with the magnet configured end-on. MP3, MP4 and MP6 could not be detected in the trachea of any of the live animals. When the rat airways were imaged after the animals were humanely killed, the particles were still not visible, even when additional volumes were added using the syringe pump. MP5, which had the highest iron oxide content, was the only visible particle and so was used for assessing and describing the in-vivo behaviour of the MP.

**Figure 7:**
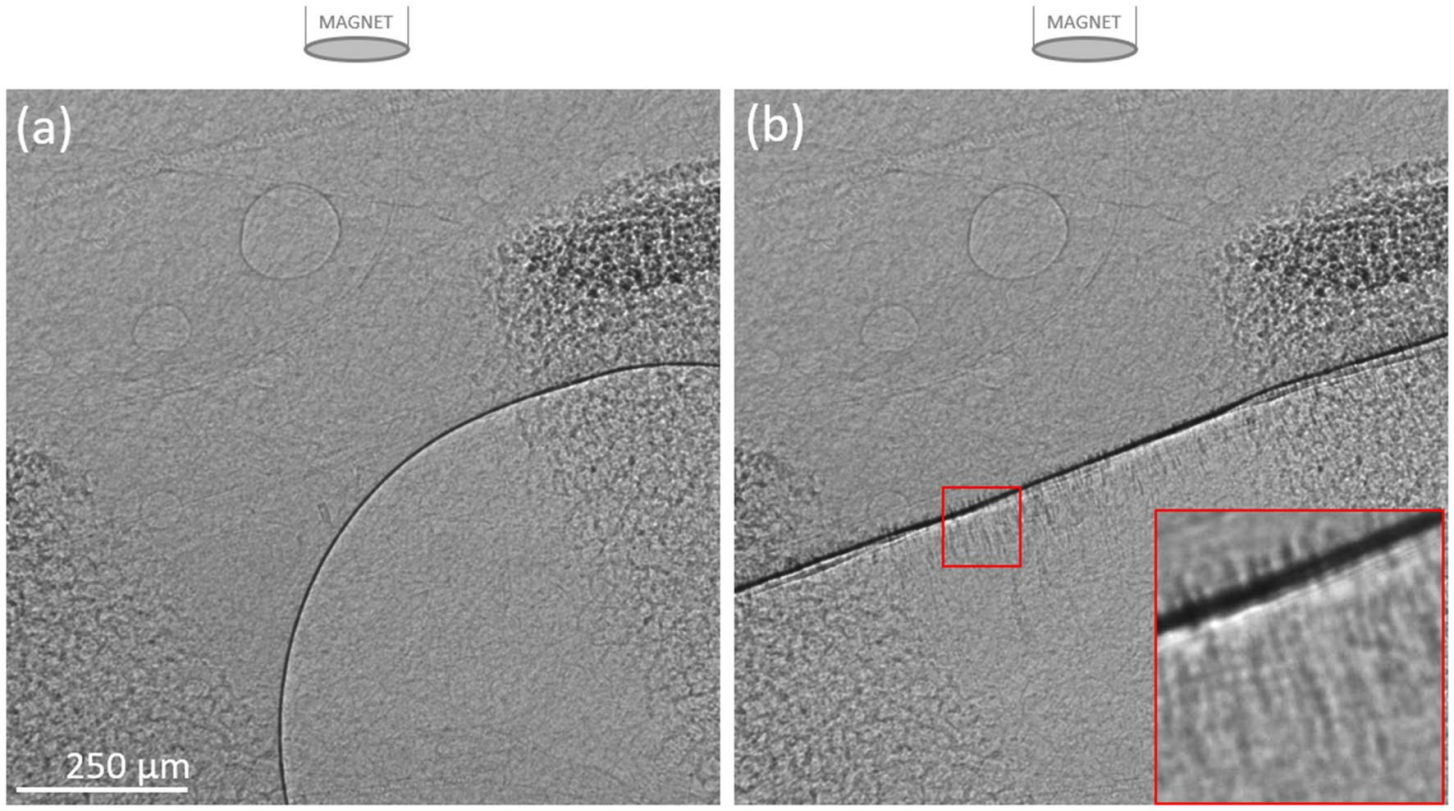
Images from (**a**) before and (**b**) immediately after delivery of MP5 into the trachea of a recently euthanized rat, with the magnet located directly above the imaging region. The imaging region is between two cartilage rings. Some fluid was present in the airway prior to MP delivery. The red box contains a contrast-enhanced enlargement. These images are from the video shown in Supplementary Video S6: MP5.

Placing the magnet above the trachea during MP delivery resulted in many but not all of the MP aggregating within the field of view. The delivery of the particles into the trachea was best observed in a humanely killed animal. Figure 7 and Supplementary Video S6: MP5 shows the rapid magnetic capture and alignment of the particles into strings on the ventral tracheal surface, demonstrating that MP could be directed to desired regions of the trachea. When searching more distally along the trachea after MP delivery, some MP were found closer to the carina, indicating that the magnetic field strength was not sufficient to collect and retain all the MP as they transited past the area of maximum magnetic field strength during the fluid delivery. Nonetheless, the post-delivery MP concentration was higher around the imaging region, suggesting that many MP were retained in the airway regions where the applied magnetic field strength was highest.

**Figure 8:**
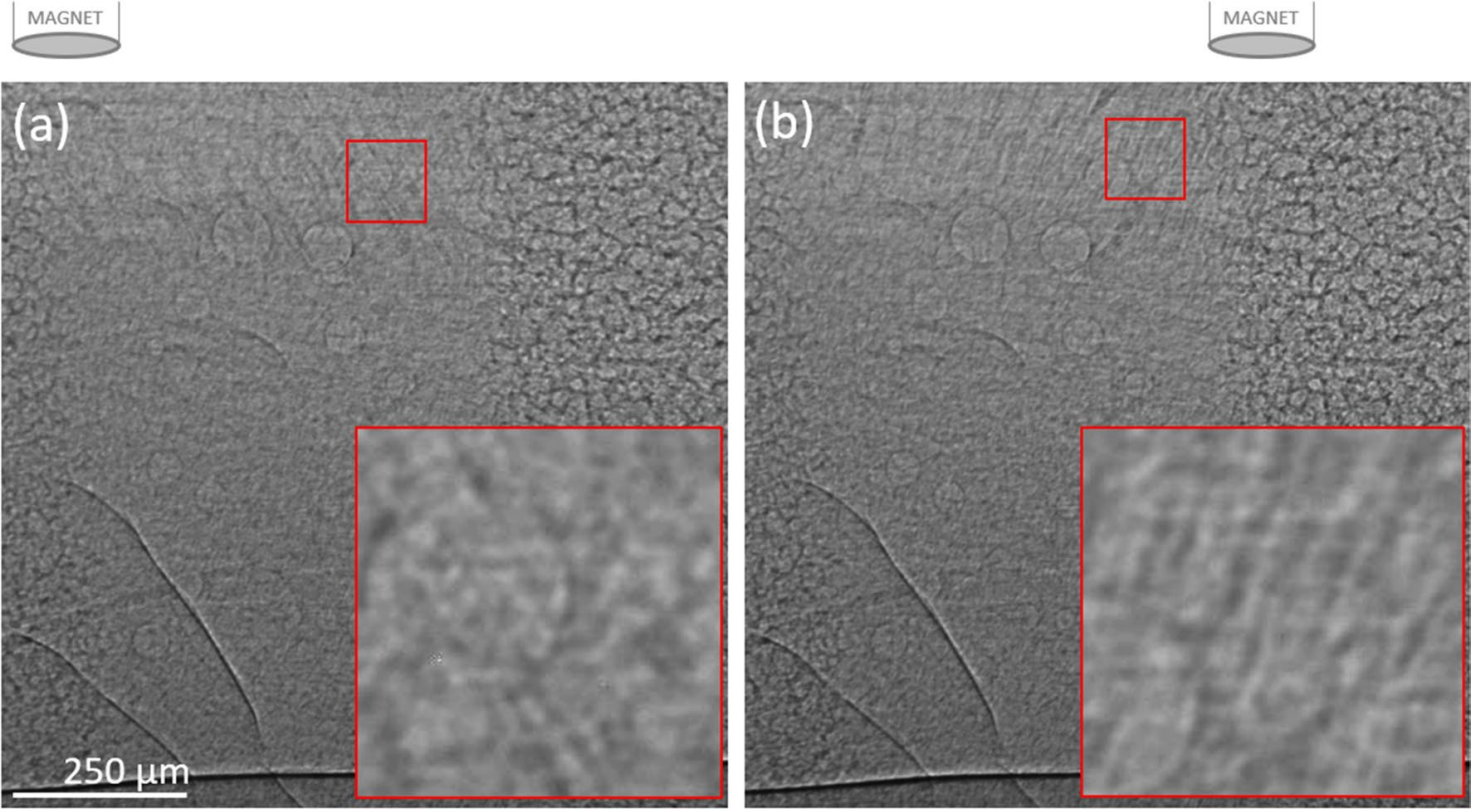
Example images showing MP5 within the trachea of a live anaesthetised rat. Images were acquired (**a**) with the magnet above and to the left of the trachea, and then (**b**) after the magnet was moved to the right. The red boxes contain contrast-enhanced enlargements. These images are from the video shown in Supplementary Video S7: MP5.

Translating the magnet along the trachea in-vivo caused the MP strands to change angle within the airway surface in a similar manner to that seen in the capillary tube (see Fig. 8 and Supplementary Video S7: MP5). However, in our studies the MP could not be dragged along the living airway surface as had been possible with the capillary tube. In some cases, the MP strands became longer as the magnet was moved left and right. Interestingly, we also found that as the magnet was translated longitudinally along the trachea the particle strings appeared to alter the depth of the layer of surface fluid, expanding it as the magnet moved directly overhead and the particle strings rotated into a vertical position (See Supplementary Video S7: MP5 at 0:09, lower right). When the magnet was translated across the top of the trachea in a transverse direction (i.e., to the left or right of the animal rather than along the length of the trachea) the characteristic pattern of motion changed. The particles were still clearly visible when moving, but the tips of the particle strings became visible when the magnet moved away from the trachea (See Supplementary Video S8: MP5, from 0:08). This was consistent with the MP behaviour we observed under applied magnetic fields in the glass capillaries.

When two magnet poles were configured north–south (i.e. to attract; Fig. 3b) above and below the trachea, the MP strings appeared longer, and were located on the side walls of the trachea rather than on the dorsal tracheal surface (See Supplementary Video S9: MP5). However, when using the two-magnet setup a high concentration of particles in a single location (i.e., the dorsal tracheal surface) was not detected after fluid delivery, as typically occurred using the single-magnet setup. When one magnet was then reversed with the poles configured to repel (Fig. 3c), the number of particles visible in the field of view did not appear to increase following delivery. Both double-magnet configurations were challenging to set up due to the high magnetic field strength pulling or pushing the magnets, respectively. The setup was then changed to be a single magnet parallel to the airway but crossing it at 90 degrees so that the field lines passed orthogonally through the wall of the trachea (Fig. 3d), an orientation designed to determine if particles could be observed aggregating on the side walls. However, no MP accumulation or motion with magnet movement was discernible with this configuration. Based on all of these results, the single-magnet, 30-degree orientation configuration (Fig. 3a) was chosen for the in-vivo gene vector studies.

When imaging was repeated immediately after the animals were humanely killed, the absence of confounding tissue motion meant that much finer and shorter lines of particles could be discerned in the clear inter-cartilage field, ‘swinging’ in concert with the translational movement of the magnet. Nonetheless, the presence and motion of the MP6 particles could still not be clearly visualised.

### in-vivo gene vector studies

The LV-LacZ titre was 1.8 × 10^8^ TU/ml, and after mixing 1:1 with CombiMag MP (MP6) animals received a 50 μl tracheal dose of 9 × 10^7^ TU/ml LV vector (i.e., 4.5 × 10^6^ TU/rat). In these studies, rather than translating the magnet during delivery we held the magnet in one location to determine whether LV transduction (a) could be improved compared to vector delivery without a magnetic field present, and (b) if airway cell transduction could be focussed to that magnetically targeted region in the upper airway.

**Figure 9:**
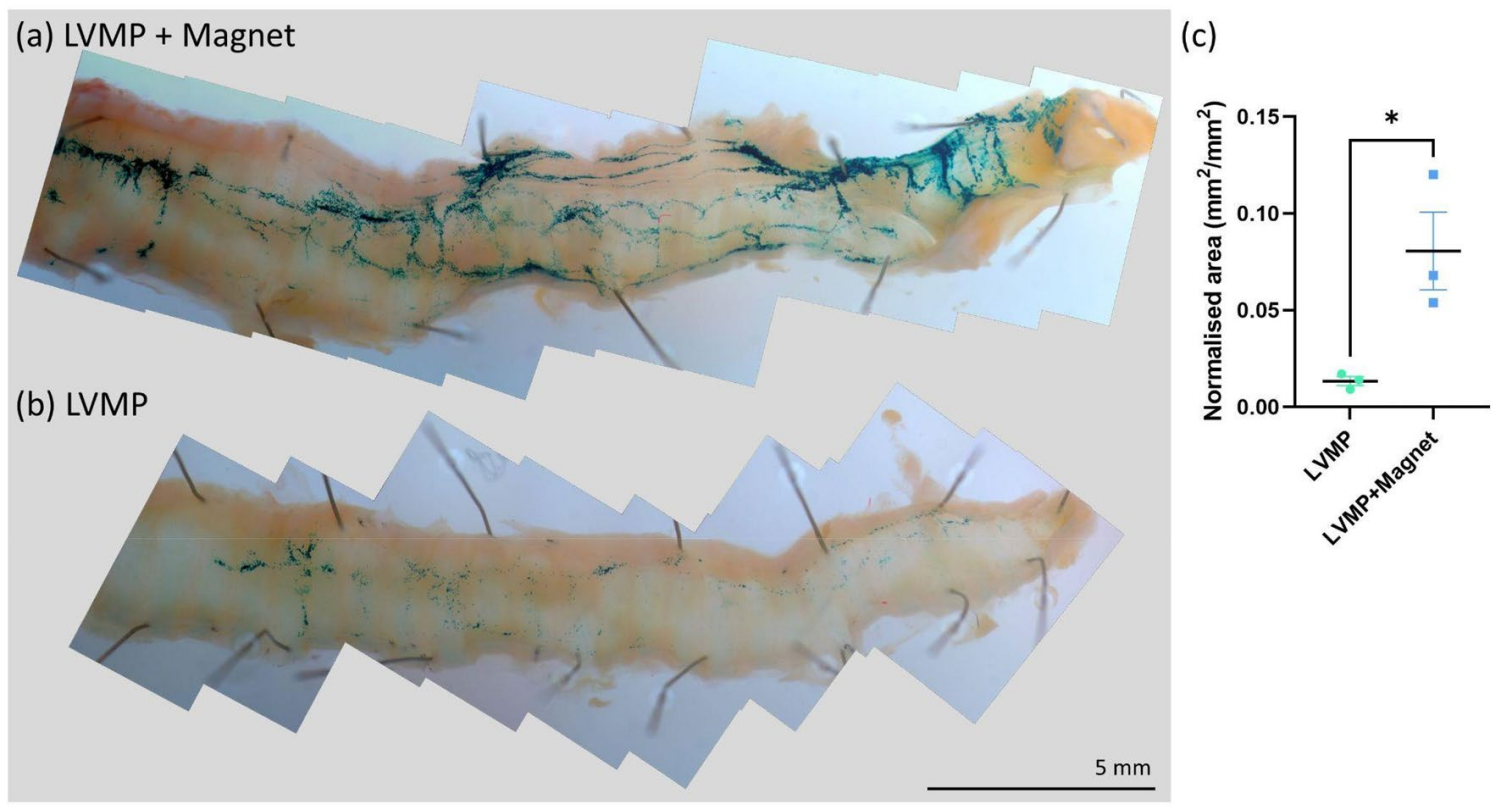
Example composite images showing the tracheal transduction produced by the LV-MP (**a**) in the presence of the magnetic field, and (**b**) without the magnet present. (**c**) There was a statistically significant improvement in the normalised LacZ transduced area within the trachea when using the magnet (*p = 0.029, t test, n = 3 per group, mean ± SEM).

The presence of the magnets and the use of CombiMag conjugated to the LV vector appeared to have no untoward effects on animal health, like our standard LV vector delivery protocols. *En face* images (Supplementary Fig. 1) of the tracheal regions that received the mechanical perturbation demonstrated that substantially higher transduction levels were present in the group of animals treated with LV-MP when the magnet was present (Fig. 9a), compared to the control group where only small amounts of blue LacZ staining were present (Fig. 9b). Quantification of the normalised X-Gal-stained area showed that LV-MP dosing in the presence of the magnetic field produced an ~ 6 × improvement (Fig. 9c).

The neutral fast red stained sections (example shown in Supplementary Fig. 2) showed LacZ stained cells were present in similar patterns and locations to those previously reported^30^.

## Discussion

A key challenge for airway gene therapies remains the accurate targeting of the vector particles to the region of interest and achieving high levels of transduction efficiency in a moving lung in the presence of airflow and active mucus clearance. For LV vectors designed to treat CF airway disease, increasing the residence time of the vector particles within the conducting airways is a goal which, to date, has been difficult to achieve. As noted by Castellani et al., the use of magnetic fields to improve transduction has advantages compared to other gene delivery approaches such as electroporation, because it could combine simplicity, cost-effectiveness, delivery localisation, improved efficiency with a smaller incubation time, and could require smaller vector doses^10^. However, the in-vivo deposition and behaviour of magnetic particles within the airways under the influence of external magnetic forces has never been described, nor indeed has the in-vivo feasibility of this approach to improve gene expression levels in intact living airways been demonstrated.

**Figure 10:**
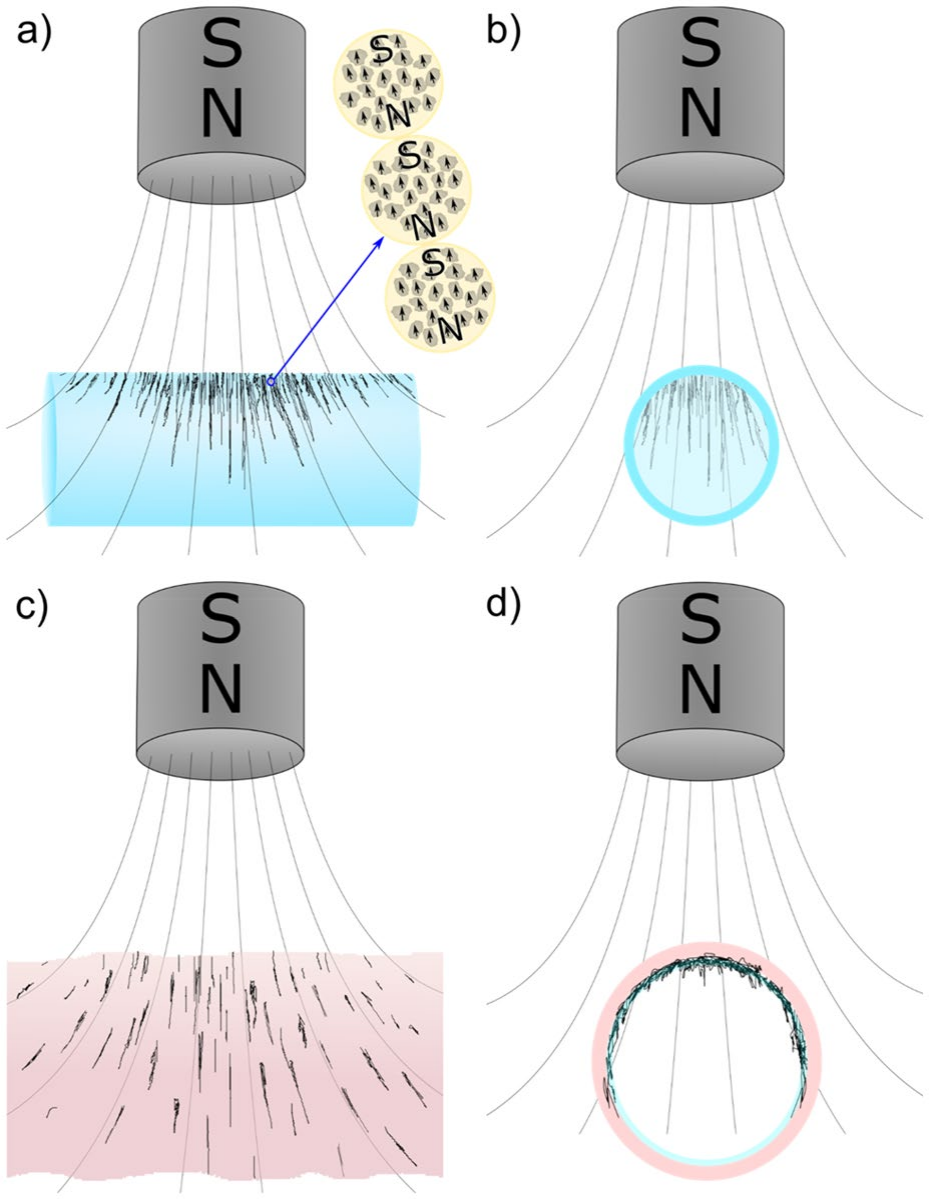
Schematic showing (**a**,**b**) the creation of particle strings within a fluid filled capillary tube and (**c**,**d**) an air-filled trachea. Note that the capillary tube and trachea are not drawn to scale. Panel (**a**) also contains a depiction of MP containing Fe_3_O_4_ particles arranged into strings.

Our in-vitro synchrotron PCXI experiments showed that all particles we tested other than the polystyrene MP were visible with the imaging setup we employed. In the presence of the magnetic field the MP formed strings, the length of which was related to the particle type and to the magnetic field strength (i.e., proximity and motion of the magnet). As depicted in Fig. 10, the strings we observed form as a result of each individual particle becoming magnetised and inducing its own local magnetic field. These individual fields cause other like-particles to aggregate and concatenate, with group string-like movement due to the local forces of local attraction and repulsion from the other particles.

When the magnet was moved above the capillary tube, the angle of the particle strings reached a critical threshold for the Fe_3_O_4_-containing MP3-5, after which the strings did not stay in their original position but moved along the surface towards the new position of the magnet. This effect likely occurred because the glass capillary surface was smooth enough to allow this movement to take place. Interestingly, MP6 (CombiMag) did not behave in this manner, potentially because the particles were smaller, had a different coating or surface charge, or the proprietary carrier fluid influenced their ability to move. The image contrast for the CombiMag particles was also weaker, suggesting that the fluid and particles may have similar densities, and as a result also did not move past each other as easily. The particles could also become stuck if the magnet moved too fast, suggesting that the magnetic field strength could not always overcome the friction between the particles in the fluid, indicating, not unsurprisingly perhaps, that magnetic field strength and the distance between the magnet and target area are important. Together these results also implied that while a magnet could capture many MP flowing past the target region, it would be very unlikely that the magnet could be relied on to move CombiMag particles along the tracheal surface. Accordingly, we concluded that the in-vivo LV-MP studies should utilise a static magnetic field to physically target a specific region of the airway tree.

When the particles were delivered in-vivo they were much harder to identify against the complex moving body-tissue background, but the ability to detect them was enhanced by translating the magnet horizontally above the trachea to make the MP strings ‘swing’. Although live imaging was possible, it was much easier to discern particle motion once the animals were humanely killed. When the magnet was in place above the imaging region, the MP concentration was typically highest in that location, although some particles were often found further along the trachea. In contrast to the in-vitro studies, particles could not be dragged along the trachea by translating the magnet. This finding is consistent with how the mucus that covers the tracheal surface normally deals with inhaled particles, trapping them onto and into the mucus for subsequent removal via mucociliary clearance mechanisms.

We hypothesised that using a magnet above and below the trachea to attract (Fig. 3b) might produce a more uniform magnetic field, rather than a field that is highly concentrated in one spot, potentially resulting in a more uniform distribution of particles. However, our preliminary studies did not find clear evidence to support this hypothesis. Likewise, configuring a pair of magnets to repel (Fig. 3c) did not result in more particle deposition in the imaging region. These two findings suggest that a two-magnet setup could not provide substantially improved local control of MP targeting, and due to the strong magnetic forces generated it was difficult to configure, making this approach less practical. Similarly, orienting the magnet above and across the trachea (Fig. 3d) also did not increase the number of particles retained in the imaging region. Some of these alternative configurations may have been unsuccessful because they resulted in a lower magnetic field strength within the deposition region. As a result, the single 30-degree angled magnet configuration (Fig. 3a) was deemed to be the simplest and most effective approach for in-vivo testing.

The LV-MP studies demonstrated that when LV vector is conjugated with CombiMag and delivered in the presence of a magnetic field following physical perturbation^28^ the transduction levels in the trachea are dramatically increased compared to the control group. Based on the synchrotron imaging studies and LacZ results, the magnetic field is clearly capable of retaining the LV within the trachea and reducing the number of vector particles that immediately move deeper into the lung. Such targeting improvements could result in higher efficacy combined with lower delivered titres, off-target transduction, inflammatory and immune side effects, and gene vector costs. Importantly, according to the manufacturer, CombiMag can be used in combination with other gene transfer approaches including with other viral vectors such as AAV, and with nucleic acids.

The primary limitation of this study was that the CombiMag (MP6) was not visible in the in-vivo imaging studies, so it was necessary to examine the effects of the various magnet strategies using MP5 instead, and assume that both behaved in a similar manner on the airway surface. Furthermore, while the in-vitro studies were designed to help predict the likely in-vivo MP behaviour, the capillary tubes were completely filled with fluid (Fig. 10b), rather than an air-filled epithelial lumen with a thin layer of surface fluid (Fig. 10c). Although this altered physical setting likely resulted in differences in behaviour between the two systems, the localization and dynamic control capabilities of magnet placement remained similar. With our two-dimensional X-ray imaging system in the in-vivo studies, it was not possible to precisely identify the particle locations and determine if they were located on the left or right wall of the trachea, on both walls, or free somewhere else within the airway lumen. Finally, all our in-vivo imaging experiments were performed with either one or two magnets already in place, as shown in Fig. 3, with the MP delivered while imaging. The use of a control group that received the MP without the magnet, as was performed for the LV-MP studies, could have verified that MP are not retained within the field of view in the absence of the magnetic field.

Since the magnet could cause the MP strings to move in-vivo, they were unlikely to be stuck in any mucus present in these normal animals immediately after delivery. Future studies should assess whether the ability to move the MP changes over time as the carrier fluid is absorbed, and whether additional fluid (potentially deposited via aerosol delivery) enhances the ability to drag the particles with the magnet. In addition, because respiratory mucus is a primary barrier to gene transfer into airway epithelial cells, particularly in the CF airway^31^, LV-MP have the potential to improve transduction levels in this environment. However, the present study was not designed to assess the effects of altered airway mucus rheology on MP penetration or LV-MP transduction levels. Future imaging studies should test MP behaviour in an appropriate CF model^32^, along with alternative MP that are capable of penetrating mucus^33^, which could be assessed to verify whether they further improve transduction levels. Finally, all these studies were performed only in the trachea. For the PB-PCXI imaging studies this was due to the inability to perform in-vivo high-magnification imaging deeper into the lung due to the increased background tissue motion during respiration and the rapid increase in tissue image complexity as the imaged area in gas exchange regions increased the depth of transit of the X-ray beam and the contrast induced by this tissue.

Future studies should examine whether the use of LV-MP and magnetic targeting alters the types of airway cells transduced. The ability to target LV-MP to deeper regions of the conducting airway tree should also be examined, including in larger animal models where the deeper conducting airways can be accessed with human bronchoscopy equipment. However, the magnetic field strength required for targeting LV-MP to the conducting airways in humans may be higher than what is achievable using the NdFeB rare-earth magnets used in this study. Our calculations suggest that sufficiently strong magnetic fields are available clinically; for example, the strength of the bar magnet is small compared to MRI magnets. For human applications, we believe large coils and a power supply could produce an appropriate magnetic field. The effect of field strength and amount of time the magnet is applied for has on transduction levels, as well as investigation of other methods for producing a desired magnetic field (e.g., electromagnets, MRI-type RF coils), should therefore also be considered in future small and large animal studies.

For some patients with CF there are few therapies available, and for most the disease results in a poor quality of life and early death. Airway genetic therapies have the potential to treat all people with CF and prevent or halt the progression of lung disease. However, accurate targeting of gene vectors to the conducting airways, and maintaining them there for the appropriate residence time, has proved challenging to-date. Here we have demonstrated the ability to control MP delivery to airway surface in-vivo and validated that this approach results in significantly higher levels of LV-MP mediated gene delivery. The MP methodologies developed in this project could ultimately be used to deliver therapeutic CFTR-containing gene vectors more efficiently compared to current methods. Our MP targeting methods, together with the combination of the appropriate gene vector and PB-PCXI visualisation technologies, provides an innovative new approach to targeted airway surface agent delivery for lung health correction.

## Supplementary Information

Supplementary Figure 1.

Supplementary Figure 2.

Supplementary Legends.

Supplementary Video 1.

Supplementary Video 2.

Supplementary Video 3.

Supplementary Video 4.

Supplementary Video 5.

Supplementary Video 6.

Supplementary Video 7.

Supplementary Video 8.

Supplementary Video 9.

## Data Availability

The PCXI data generated in this study is available from the authors on reasonable request.
