# Supplementary figures and images for: Improved in-vivo airway gene transfer via magnetic-guidance, with protocol development informed by synchrotron imaging
Source: Sci Rep. 2022 May 30;12:9000. doi: 10.1038/s41598-022-12895-x (PMC9151774; doi:10.1038/s41598-022-12895-x)

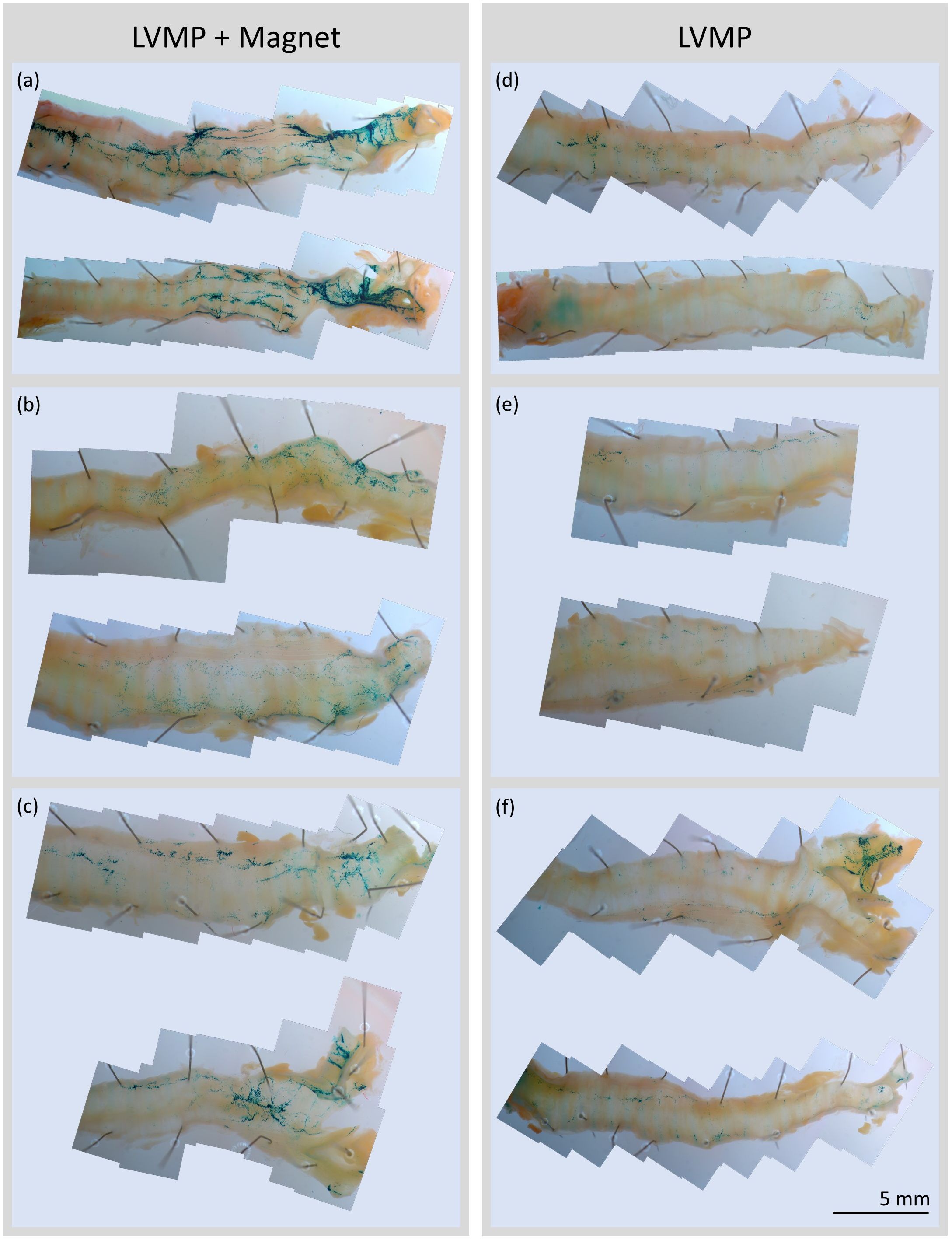

Supplement: Supplementary file 1 — Supplementary Figure 1. [file 41598_2022_12895_MOESM1_ESM.jpg]

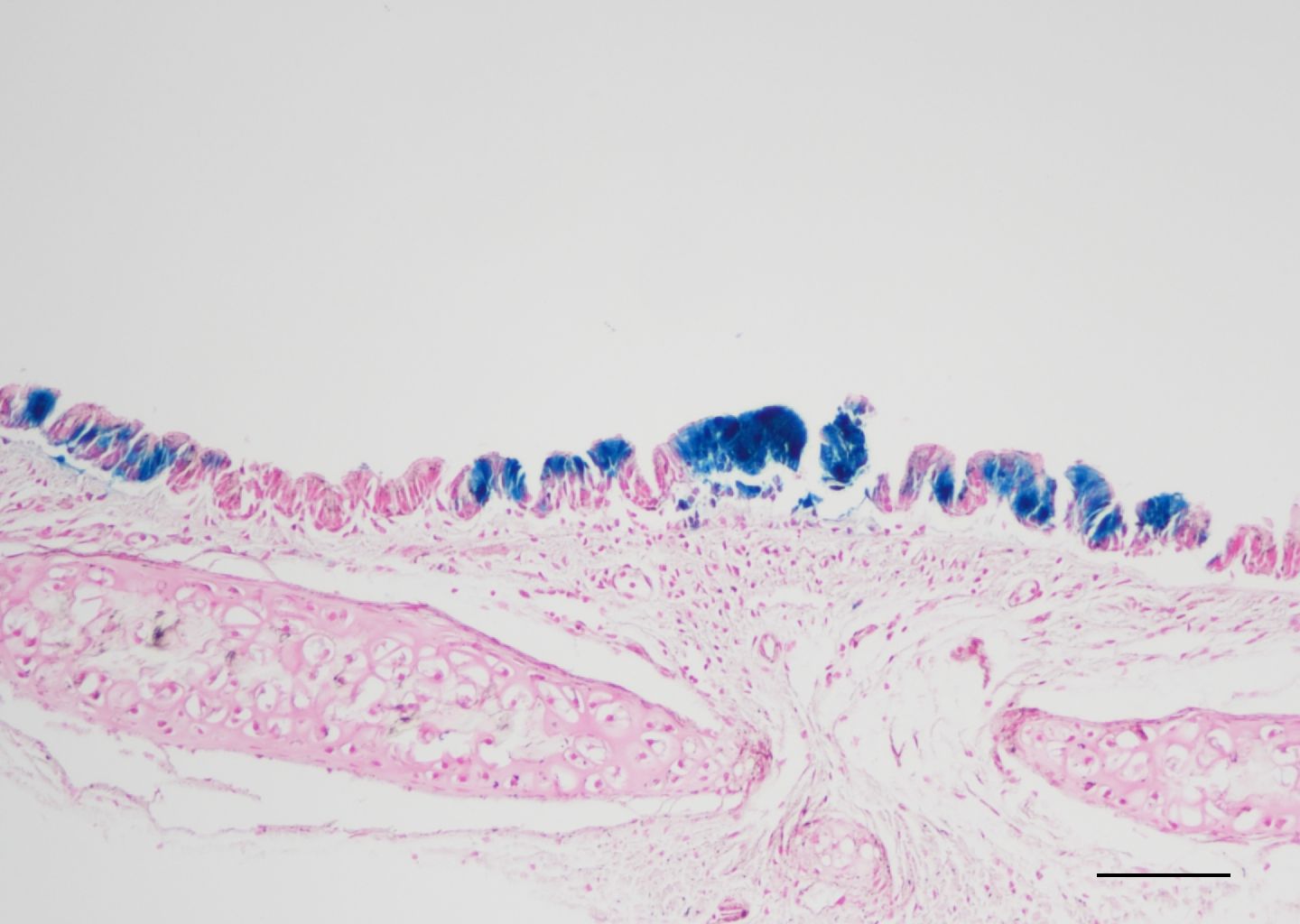

Supplement: Supplementary file 2 — Supplementary Figure 2. [file 41598_2022_12895_MOESM2_ESM.jpg]
