# Supplementary material for: Improved in-vivo airway gene transfer via magnetic-guidance, with protocol development informed by synchrotron imaging
Source: Sci Rep. 2022 May 30;12:9000. doi: 10.1038/s41598-022-12895-x (PMC9151774; doi:10.1038/s41598-022-12895-x)
Supplement: Supplementary file 3 — Supplementary Legends. [file 41598_2022_12895_MOESM3_ESM.docx]

## Supplementary material captions

## Supplementary Figure 1

Composite images from all animals showing the transduction (blue stained cells) produced by the LV-MP (a-c) in the presence of the magnetic field, and (d-f) without the magnet present. The trachea from each animal was cut open, so both halves are shown. Left is the caudal end of the trachea.

## Supplementary Figure 2

Neutral fast red stained image showing LacZ transduced cells (blue staining) in the trachea of a rat that received LV-MP in the presence of a magnetic field. Scale bar 250 μm.

## Supplementary Video S1

This video shows the *in vitro* behaviour of MP4 in a capillary tube as the magnet was brought closer from above. This increases the magnetic field strength, shortening the particle strings, and causing the particles to migrate toward the top surface of the capillary, increasing the particle packing density. The tube also inadvertently contains air bubbles, which exhibit strong phase contrast edge effects, and affect motion of the particle strings.

## Supplementary Video S2: MP4

When the magnet is moved away from the capillary tube the magnetic field strength decreases, causing the MP4 particle strings to increase in length. The MP strings appear to release trapped debris as they move (e.g., left of large bubble). Note that this is the same capillary as Supplementary Video S1.

## Supplementary Video S3: MP5

MP5 was the most visible particle in the *in vitro* capillary testing system, with long dense strings that extend almost all the way across the tube. Some strings appear to not be in contact with the upper surface; this is due to the visualisation of the cylindrical tube in two dimensions, with the string bases being positioned a small distance away on either side from the top of the capillary. The magnet begins centred above the tube and is then moved to the right. Particles to the left of the field of view are pulled across to the right, sweeping the region clear.

## Supplementary Video S4: MP6

MP6 (CombiMag) was weakly visible in the capillary tube. When the magnet was moved in the same manner as for Supplementary Video S3, the MP6 strings changed angle, but no strings were swept across the field of view.

## Supplementary Video S5: MP3

When the magnet was moved away from the capillary tube (up and to the left) and the magnetic field strength decreased, the MP3 particles in the field of view dropped to the bottom of the capillary tube under gravity. Interestingly, they remained in strings. Note that the imaging location is the right-hand end of the capillary tube, and some strings can be seen falling across the spherical face of the end bubble held in by plug material.

## Supplementary Video S6: MP5

For all in vivo videos the rats are supine with the mouth to the right and lungs to the left, and the videos are 15-25x normal speed depending on respiratory rate. The solid black line is the interface between fluid and air, with the white edge on the luminal side. This video was taken several minutes after the rat was humanely killed while on the imaging stage and showed substantially less background movement compared to the live imaging in Supplementary videos S7 and S9. MP5 was delivered during imaging and demonstrates that the particles can be rapidly captured onto the airway surface when delivery occurs in the presence of a magnetic field. The video starts with the trachea partly fluid filled with a large air bubble on the bottom right. The MP5 particles enter from the right, depositing as strings as a result of the magnetic field as the injected fluid passes this region, leaving an air-filled trachea.

## Supplementary Video S7: MP5

*In vivo* video of the trachea of a live anaesthetised rat at four locations within the airway (proximal ventral, distal ventral, distal dorsal and proximal dorsal). The airway surface is clearly identifiable by the strong black/white lines, and cartilage rings have a regularly spaced pavement-like pattern. Note that this imaging setup is a two-dimensional projection through a range of tissues, so features such as surface skin folds (e.g., at 7 seconds) are apparent. Long strings of MP5 are visible in the centre of the airway and move as the magnet is translated overhead to the left and right of the imaging plane. At the final location, the surface fluid containing MP moves in concert with the movement of the magnet.

## Supplementary Video S8: MP5

This video is an image sequence acquired shortly after the video in Supplementary videos S6. MP5 particles are present on the dorsal airway surface and the strings moved as the magnet was translated to the right and then back to the left. The magnet was then returned to above the field of view (at 6-7 seconds), and then translated across and above the trachea (i.e., into and out of the field of view) in a direction orthogonal to the previous motion. This changed the motion and view of the strings to be end-on.

## Supplementary Video S9: MP5

When two magnets were placed above and below the trachea of a live anaesthetised animal and configured to attract, the MP5 particle deposition was different to with the single magnet. The resulting strings were longer and appeared to be primarily located on the side walls of the trachea (i.e., the centre of the field of view), rather than on the dorsal tracheal surface. When the magnet above the trachea was translated to the right and left, the strings, which covered the entire field of view, moved in response to the magnet. Note that the central air bubble was also moved with the MP5 strings.
